# From Syndemic Lesson after COVID-19 Pandemic to a “Systemic Clinical Risk Management” Proposal in the Perspective of the Ethics of Job Well Done

**DOI:** 10.3390/ijerph19010015

**Published:** 2021-12-21

**Authors:** Francesco De Micco, Anna De Benedictis, Vittorio Fineschi, Paola Frati, Massimo Ciccozzi, Leandro Pecchia, Rossana Alloni, Nicola Petrosillo, Simonetta Filippi, Giampaolo Ghilardi, Laura Leondina Campanozzi, Vittoradolfo Tambone

**Affiliations:** 1 Bioethics and Humanities Research Unit, Campus Bio-Medico University of Rome, 00128 Rome, Italy; f.demicco@unicampus.it (F.D.M.); r.alloni@unicampus.it (R.A.); g.ghilardi@unicampus.it (G.G.); l.campanozzi@unicampus.it (L.L.C.); v.tambone@unicampus.it (V.T.); 2 Department of Anatomical, Histological, Forensic and Orthopaedic Sciences (SAIMLAL), Sapienza University of Rome, 00185 Rome, Italy; vittorio.fineschi@uniroma1.it (V.F.); paola.frati@uniroma1.it (P.F.); 3 Medical Statistics and Molecular Epidemiology Research Unit, Campus Bio-Medico University of Rome, 00128 Rome, Italy; m.ciccozzi@unicampus.it; 4 Applied Biomedical Signal Processing and Intelligent eHealth Lab, University of Warwick (UK), Warwick CV4 7AL, UK; L.Pecchia@warwick.ac.uk; 5 Campus Bio-Medico University Hospital, 00128 Rome, Italy; n.petrosillo@unicampus.it; 6 Non Linear Physics and Mathematical Models Research Unit, Campus Bio-Medico University of Rome, 00128 Rome, Italy; s.filippi@unicampus.it

**Keywords:** clinical risk management, enabling technologies, big data, quality of care, patient safety, sustainability, support for policy making, medical ethics, Bayesian network

## Abstract

The syndemic framework proposed by the 2021–2030 World Health Organization (WHO) action plan for patient safety and the introduction of enabling technologies in health services involve a more effective interpretation of the data to understand causation. Based on the Systemic Theory, this communication proposes the “Systemic Clinical Risk Management” (SCRM) to improve the Quality of Care and Patient Safety. This is a new Clinical Risk Management model capable of developing the ability to observe and synthesize different elements in ways that lead to in-depth interventions to achieve solutions aligned with the sustainable development of health services. In order to avoid uncontrolled decision-making related to the use of enabling technologies, we devised an internal Learning Algorithm Risk Management (LARM) level based on a Bayesian approach. Moreover, according to the ethics of Job Well Done, the SCRM, instead of giving an opinion on events that have already occurred, proposes a bioethical co-working because it suggests the best way to act from a scientific point of view.

It is estimated that one in every 10 patients is harmed while receiving hospital care and patient harm exerts a considerable global health burden. According to OECD, 15% of total hospital activity and expenditure is a direct result of adverse events [1].

Medication and diagnostic errors, hospital infections, unsafe surgical care procedures and inappropriate or unskilled use of medical radiation are the leading cause of avoidable harm in health care around the world. Any one or a combination of these can result in severe patient harm, disability and even death [2].

Health Care-Associated Infections (HCAIs) occur in 7 out of 100 hospitalized patients in high-income countries and in 10 out of 100 hospitalized patients in low-and middle-income countries [3]. In Europe, HCAIs caused 16.4 million extra hospital days a year and approximately 37,000 deaths [4,5]. In U.S., the estimated number of HCAIs was approximately 1.7 million and HCAIs-associated deaths were around 98,987 [6].

Among hospitalized patients, falls are the most frequently reported safety incident in hospitals [7]. Fall rates range from 3 to 11 falls per 1000 bed days [8]. Yet falls are one of the most difficult patient safety issues to address because of their many causes.

Over the past year, the COVID-19 pandemic has further compromised patient safety and new recommendations have had to be made [9]. Facing the COVID-19 pandemic, several ethical debates have arisen such as patient triage [10].

Clinical Risk Management specifically is concerned with improving the quality and safety of healthcare services by identifying the circumstances and opportunities that put patients at risk of harm and then acting to prevent or control those risks [11,12]. Good clinical risk management has a dual projection: individual, regarding patient safety; collective, considering socio-cultural, political, organisational and economic consequences [12].

Within this broad debate, WHO provided a multi-disciplinary approach based on: (a) implementation of protective legislative measures including safety and accreditation standards; (b) building of highly reliable health systems characterized by good governance, transparency and a no blame culture; (c) safety of clinical processes; (d) patient and family engagement; (e) identification of centers of excellence in patient safety education and training; (f) development and support of multi-sectoral and multinational synergies, partnerships and solidarity; (g) implementation of Clinical Risk Management (CRM) activities. Regarding the latter strategic objective, it is mandatory to identify a responsible team in each health facility and increase the reporting systems in real time to better understand the probable causal link [13].

In our opinion, WHO Patient Safety approach to prevent and reduce risks during provision of health care reflects a Syndemic framework.

According to the series published by “The Lancet”, the Syndemic model of health emphasizes the relationships between diseases or health conditions and the social, economic, environmental, and political milieu in which a population is immersed. Political and economic changes can cause breakdowns in health measures resulting in differential and additive harmful effects on specific populations [14].

The WHO proposal aims to enhance quality of care and patient safety by considering regulatory aspects, clinical governance, patients and their families, and multisectoral and multinational synergies.

In our opinion, WHO Patient Safety approach to prevent and reduce risks during provision of health care reflects a Syndemic framework. “A syndemics framework exam-ines the health consequences of identifiable disease interactions and the social, environ-mental, or economic factors that promote such interaction and worsen disease” [15]. Clinical risk management based on a syndemic approach means recognizing the influence that legislative measures, hospital governance, data transparency, family involvement, the medical education system as well as multisectoral and multinational synergies have on patient safety.

The strategic objectives recommended by the WHO produce a significant amount of data. The recognition of probable causal link is the outcome of a complex multifactorial analysis based on the impact of social, environmental, or economic factors on health.

For this reason, we propose a stronger foundation of the Syndemic approach in the Systemic Theory [16] because it is necessary to implement effective CRM in complex situations. CRM requires a systemic approach, understood as “The theoretical and practical ability to observe […] and synthetize components, functions, connections, structures, interrelationships, and dynamics across disciplines, functions, organizations, people, trends, and cultures in ways that lead to insightful problem interventions for attaining solutions aligned with sustainable development” [17].

To integrate the methods of natural and social sciences, general systems theory aims to describe the laws of totality in contrast to the mechanism of classical science and the principle of one-way causality [18]. In a complex system, the behavior of the whole is more complex than the behavior of the part and the relationships may be non-linear and the effects multi-causal [19].

In the theory of causality, complexity assumes an important role [20]. The awareness of the limitations of each individual approach imposes the duty to make the research open to other contributions that bring knowledge from mono to multi-dimensional and, therefore, closer to the complex nature of the phenomenon under study [21].

Enabling technologies, such as Artificial Intelligence (AI), the Internet of Things, Robots and Big Data, seem to be very effective in supporting public health interventions. However, creating a new health system through the use of Enabling technologies requires change on a profound scale [22,23,24,25,26]. Yet, their introduction requires the organization of NHS services to maintain healthcare services sustainability. Health-enabling technologies may lead to both new ways of living and new ways of health care. Both ways are interwoven [27].

The introduction of those enabling technologies and the consequent requirement for a reorganization of healthcare services determine an urgent need to apply systematic frameworks to improve the quality and safety of health-care services. Furthermore, it entails going beyond the application of traditional data correlation, enforcing more effective interpretation for understanding causation links.

Correlation of multifactorial e-tech data and human interpretation of data is what we define “Systemic Clinical Risk Management” (SCRM).

The Clinical Risk Management activity is developed through a “reactive approach” based on the knowledge and analysis of the error, the identification and correction of the causes of the error and the monitoring of the measures implemented to prevent the error [28].

Systemic Clinical Risk Management (SCRM) is a ‘proactive approach’ based on the acquisition and processing of quantitative and qualitative data (e.g., living conditions, economic, behavioral, food, etc.). The COVID-19 pandemic taught us that the qualitative dimension of a person’s health can be derived from the human-animal-environmental interface, lifestyle behaviors, social factors, political and socio-economic conditions and globalization processes [29]. The use of enabling technologies should help us to develop a proactive approach to patient safety, including the qualitative dimension.

We are convinced that this “Systemic Risk Prevention” will improve: (a) the quality of care and patient safety; (b) the efficiency of Public Health System; (c) the decrease in legal litigation; (d) the confidence in technologies; (e) the collaboration between science and politics based on evidence.

The awareness of the limitations of each individual approach imposes the duty to make the research open to other contributions that bring knowledge from mono to multidimensional and, therefore, closer to the complex nature of the phenomenon under study. Scientific knowledge and consequent action, in other words, is a “collective approach”.

“Reduction” is also useful and necessary for medicine that wants to be scientific and increasingly effective, but requires in order being conducted correctly, specific logical expedients, such as the use of non-linear logic [30], systemic correlations [31] and multidimensional observation. The application of these devices bears results at different levels of Public Health intervention in the area of patient safety and quality of care.

According to WHO’s guidance on AI ethics and governance for health, SCRM puts ethics and human rights at the heart of its design, deployment, and use [32].

According to the ethics of Job Well Done, we argue that the SCRM in the context of realist/objective methodology consists of the desired object in the circumstance of the complete human act, without necessarily touching on the intentional aspect of the act. In this perspective, we have the following progression: point 1: the action planned and foreseen by the subject; point 2: the action desired for a reason; point 3: the desired action, for this reason, will be carried out by determined means, through a determined collaboration, at a determined time, in a determined place, etc.; point 4: the complete human act will be evaluated as a synthesis of the evaluations in points 1, 2 and 3 [33]. The SCRM instead of giving an opinion on events that have already occurred (consequentialism), proposes a bioethical co-working because it suggests the best way to act from a scientific point of view [21,34].

However, we foresee the use of enabling technologies could lead to an uncontrolled decision-making process. This is a bias that a health system cannot afford. Transparency between clinicians and patients, among clinicians and health care organizations, and between health care organizations and the public is essential for quality, safety, accountability, and informed decision making [35].

For these reasons, the question of how to develop a socially robust level of trust in new technologies increasingly assumes the connotation of a prerequisite for innovation.

For this, SCRM requires an internal level of “Learning Algorithm Risk Management (LARM)” that AI could elaborate in the meantime that builds itself as machine learning. The goals of LARM will necessarily evolve in parallel with algorithms in use. This second/internal level of SCRM suggests using Bayesian network to manage the Whole System and not only for its pre-training.

At the international level, several initiatives have already appeared with the aim of promoting a conscious development of new technologies, including the development by the European Commission of guidelines for reliable artificial intelligence in a perspective that we can define as “human centric” [4,5]. The European Parliament in a recent resolution also explicitly stated the need to “build trust in artificial intelligence, robotics and related technologies, ensuring that these are developed, disseminated and used ethically” [36].

Thomas Kuhn argued that science evolves through what he calls “paradigm shifts”. To make progress, however, you need to change your point of view and look at things in a new way. Only then, you will be ready to do better [37].

Patient safety is the cornerstone of high-quality health care [38]. In order to increase Patient Safety and the Quality of health care, we propose a specific paradigm capable of implementing Hippocratic Scientific Medicine through New Technologies.

## Data Availability

Not applicable.
